# Short-Term Association of Air Pollutant Levels and Hospital Admissions for Stroke and Effect Modification by Apparent Temperature: Evidence From Shanghai, China

**DOI:** 10.3389/fpubh.2021.716153

**Published:** 2021-09-27

**Authors:** Lvkan Weng, Na Li, Tienan Feng, Rongjia Zhu, Zhi-Jie Zheng

**Affiliations:** ^1^Department of Epidemiology and Biostatistics, School of Public Health, Shanghai Jiao Tong University, Shanghai, China; ^2^Shanghai Chest Hospital, Shanghai, China; ^3^Department of Global Health, School of Public Health, Peking University, Beijing, China; ^4^Clinic Research Center, Shanghai Jiao Tong University School of Medicine, Shanghai, China

**Keywords:** air pollution, stroke, hospital admissions, apparent temperature, generalized additive model (GAM)

## Abstract

The epidemiological evidence on relationships between air pollution, temperature, and stroke remains inconclusive. Limited evidence is available for the effect modification by apparent temperature, an indicator reflecting reactions to the thermal environment, on short-term associations between air pollution and hospital admissions for stroke. We used a generalized additive model with Poisson regression to estimate the relative risk (RR) of stroke admissions in Shanghai, China, between 2014 and 2016 associated with air pollutants, with subgroup analyses by age, sex, apparent temperature, and season. During the study period, changes in the daily number of stroke admissions per 10 μg/m^3^ increase in nitrogen dioxide (at lags 0, 1, 0–1, and 0–2) ranged from 1.05 (95% CI: 0.82%, 2.88%) to 2.24% (95% CI: 0.84%, 3.65%). For each 10 μg/m^3^ increase in sulfur dioxide concentrations at lags 1, 2, 0–1, and 0–2, the RR of daily stroke admissions increased by 3.34 (95% CI: 0.955%, 5.79%), 0.32 (95% CI: −1.97%, 2.67%), 3.33 (95% CI: 0.38%, 6.37%), and 2.86% (95% CI: −0.45%, 6.28%), respectively. The associations of same-day exposure to nitrogen dioxide with stroke admissions remained significant after adjustment for ozone levels. These associations were not modified by sex, age, apparent temperature, or season. More research is warranted to determine whether apparent temperature modifies the associations between air pollution and stroke admissions.

## Introduction

Stroke is an important driver of the growing global disease burden and the second largest contributor to disability-adjusted life lost years in individuals aged over 50 years (1). China has the highest estimated lifetime risk of stroke (39.3%; 95% CI: 37.5%, 41.4%) (2), and a considerable increase in mortality and the prevalence and incidence of stroke have been observed in recent years, of which 69.6% were ischemic stroke and 23.8% were hemorrhagic stroke (3). In addition to genetic and lifestyle factors, adverse environmental factors such as air pollution and extreme temperatures have been identified as risk factors for stroke. The association of air pollution exposure with stroke incidence has been analyzed but with divergent findings by pollutant (4–6). Moreover, studies from low- and middle-income countries are relatively scarce, although the incidence and prevalence of stroke are higher in these regions (7) and air pollution is worse.

In addition, most prior reports have focused on cerebrovascular disease or ischemic stroke, while some studies examined ischemic and hemorrhagic stroke separately but reported inconsistent results (8). There are relatively few studies on hemorrhagic stroke, and the evidence remains equivocal (5, 9, 10). Climate change is another environmental concern with public health implications, in particular, for cardiovascular and cerebrovascular events (11, 12). Several studies have shown that both higher levels of air pollution and nonoptimum temperature are associated with a higher incidence of stroke (4, 5, 13–15), and other studies have suggested a possible modifying effect of temperature in associations between air pollution and stroke (11, 16). Nonetheless, these studies showed conflicting results as to whether lower temperatures or higher temperatures could enhance the effects. In addition to ambient temperature, natural factors such as relative humidity, air pressure, precipitation, and wind speed could have a significant effect on the pattern of air pollution in China (8, 17). It might be, therefore, imperative to examine the modifying effect of a biometeorological index combining several meteorological factors. Apparent temperature (AT) combined ambient temperature, humidity, and wind speed, which can be employed to evaluate the human body reactions to various thermal environments and represent the actual human perception of ambient temperature. Some studies have explored the effects of AT on several health outcomes; nevertheless, only limited evidence exists for the effect modification of AT in air pollution-stroke associations (18).

In this study, we analyzed the associations between five air pollutants (particulate matter with a diameter ≤ 10 μm [PM_10_], particulate matter with a diameter ≤ 2.5 μm [PM_2.5_], sulfur dioxide [SO_2_], nitrogen dioxide [NO_2_], and ozone [O_3_]) and hospital admissions for stroke in Shanghai, China, between January 2014 and December 2016. The study also examined whether the association differed by stroke subtype, patient age and sex, and AT.

## Materials and Methods

### Data on Hospital Admissions

The Shanghai Municipality is located in eastern China, between 120°52′-122°12′ E longitude and 30°40′-31°53′ N latitude, with a subtropical monsoon climate. Shanghai is the largest city in China and one of the most developed cities in the world as well. Shanghai has become a global financial center and a transport hub with the busiest container port in the world. Industrial production, heavy urban traffic, and a massive increase in urban population have aggravated the emission of air pollutants (19).

**Figure 1:**
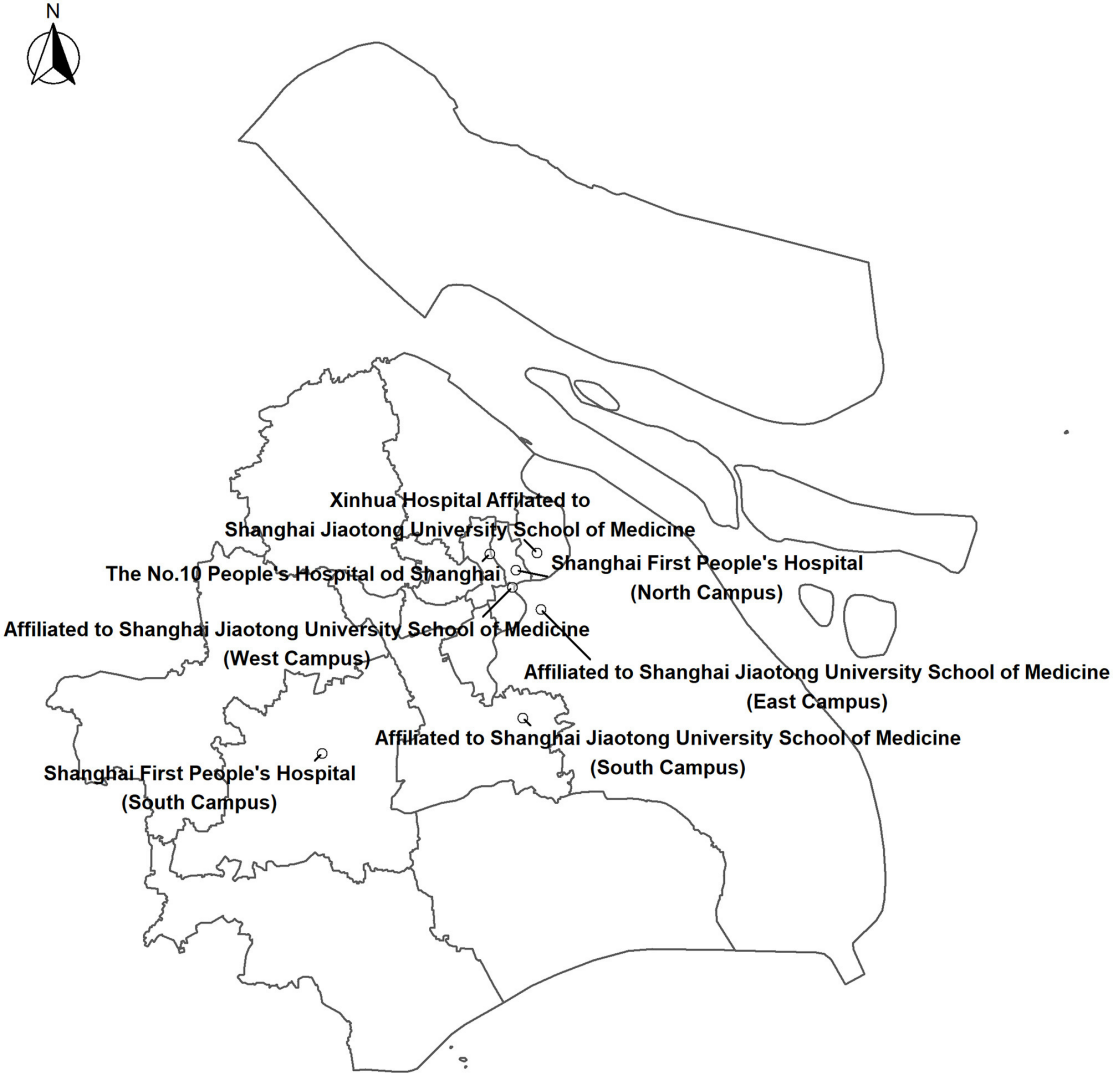
Locations of four tertiary hospitals in Shanghai.

Data on daily stroke admissions during the study period of January 2014–December 2016 were obtained from four tertiary general hospitals with the capacity to treat patients with stroke (Shanghai First People's Hospital, The No. 10 People's Hospital of Shanghai, Renji Hospital Affiliated to Shanghai Jiao Tong University School of Medicine, and Xinhua Hospital Affiliated to Shanghai Jiao Tong University School of Medicine). These hospitals have made achievements in treating neurological disease, especially stroke, and all four hospitals have widely distributed branches in urban and suburban areas covering most districts in Shanghai (Figure 1). We collected eligible cases by reviewing the admission records and electronic medical records of the neurology department at each hospital. For this study, the date of admission, sex, age, and discharge diagnosis were extracted from the records. We identified the cause of hospital admission by the International Classification of Diseases, 10th Revision (ICD-10) code of the discharge diagnosis: total stroke (ICD-10: I60–I64), ischemic stroke (ICD-10: I63), intracerebral hemorrhage stroke (ICD-10: I61), and subarachnoid hemorrhage (ICD-10: I60). We excluded stroke cases caused by trauma, tumor, abnormal coagulation function, or infection, patients who were admitted again within 28 days, cases with missing information, and those who were not permanent residents of Shanghai.

### Air Pollution and Meteorological Data

Data on concentrations of PM_10_, PM_2.5_, SO_2_, NO_2_, and O_3_ were obtained from the National Air Pollution Monitoring System. Ten national monitoring stations are located in eight districts in Shanghai. We derived daily 24-h average concentrations of PM_2.5_, SO_2_, and NO_2_ and maximum 8-h average concentrations of O_3_, averaged across all valid monitoring sites, to represent the population exposure to ambient air pollution. We obtained daily mean levels of PM_2.5_, PM_10_, SO_2_, NO_2_, and maximum daily 8-h mean O_3_ concentrations averaged across the monitors. The daily mean concentration of air pollutants for each district was calculated by averaging the concentrations of all stations in that district. A series of standards or regulations exist for the locations of monitors and the monitoring process of air pollutants by the Chinese government to ensure that the monitoring measurements reflect the air pollution levels of the urban background (20). The monitoring data have been extensively used as a proxy for population exposure to air pollution in China (21). All measurement procedures complied with Ambient Air Quality Standards of China (GB3095-2012). Meteorological data, namely, daily mean temperature, relative humidity, wind speed, rainfall, and vapor pressure, were obtained from the Shanghai Meteorological Information Center, China Meteorological Bureau. The data are collected from the Shanghai Observatory (Station No. 54511) in Xuhui District, one of the national essential meteorological stations.

### Calculation of Apparent Temperature

The AT was calculated by daily mean temperature, relative humidity, and barometric pressure using the following equations (18, 22, 23):


(1) $$\begin{array}{r} Apparent\;temperature=T+0.33\times e-0.70\times WS-4.00, \end{array}$$

(2) $$\begin{array}{r} e=\frac{relative\;humidity}{100}\times 6.105\times \exp\left[17.27\times \frac{T}{\left(237.7+T\right)}\right], \end{array}$$

where *T, e*, and *WS* denote the daily mean temperature, water vapor pressure, and average wind velocity, respectively. The water vapor pressure *e* is calculated from Equation 2 using the daily mean temperature and relative humidity.

### Statistical Analysis

A generalized additive model with Poisson regression was used to estimate the RR of stroke admissions associated with air pollutants. The potential confounding covariates were incorporated into the model, namely, temperature, relative humidity, public holidays, and day of the week, and were predefined by previously published studies. The potential confounding effects of weather, seasonality, and long-term patterns were controlled by smoothing functions (natural cubic splines). Specifically, we introduced the following covariates into the models: (1) a natural cubic spline smoother of calendar day with 7 degrees of freedom (*df*) per year; (2) natural cubic spline smoothers of the temperature on the same day as admission with 3 *df*; (3) natural cubic spline smoothers of the relative humidity with 3 *df*; and (4) indicator variables for the day of the week and public holidays. The *df* values for a calendar day, temperature, and relative humidity were selected by the parameters used in the previous studies (21, 24, 25). Consequently, the regression model was constructed as follows:


$$\begin{array}{r} \log\left[E\left(Y_{t}\right)\right]=\alpha +\beta \left(air\;pollutants\right)\\ +ns\left(calendar\;time,df=7\;per\;year\right)+ns\left(temperature,df=3\right)\\ +ns\left(relative\;humidity,df=3\right)+day\;of\;the\;week \end{array}$$

(3) $$\begin{array}{r} +public\;holidays, \end{array}$$

where *E*(*Y*_*t*_) is the expected count of admissions for ischemic stroke on day *t*; β represents the log-RR of ischemic stroke associated with a unit increase in air pollutant levels, and *ns*() indicates natural cubic spline function. Public holidays and days of the week were included in the model as indicator variables, and temperature and relative humidity indicate current-day air temperature and relative humidity, respectively. We used same-day air pollutant concentrations (lag 0) in our main analyses because lag 0 often produces the largest effect estimates (4, 17, 26, 27).

To investigate the lag effect associated with air pollutants, we used the following lag periods: single-day lags (the same day [lag 0], the previous day [lag 1], and the day before the previous day [lag 2]) and multiday lags (average concentration of the same day and previous day [lag 0–1], and average concentration of the same day and previous two days [lag 0–2]). Subgroup analyses were performed to examine whether the association differed by age (<65 years and ≥65 years), sex, AT (cool days: ≤ 19.6°C, warm days: >19.6°C; and median temperature used as the cutoff), and season (April–September, warm season; October–March, cool season). We used a *Z* test to compare differences in the association between subgroups (28). In addition to the single-pollutant model, we also assessed potential confounding by other pollutants by establishing a series of two-pollutant models. To avoid collinearity caused by high correlativity between pollutants, we incorporated O_3_ into the two-pollutant model using Spearman's correlation coefficients between air pollutants.

All results are reported as percentage changes and 95% CIs in daily hospital admissions for ischemic stroke, in association with increases of 10 μg/m^3^ in the levels of PM_2.5_, SO_2_, NO_2_, and O_3_. All analyses were conducted in R version 4.0.3 (R Foundation for Statistical Computing, Vienna, Austria).

### Ethical Approval

This study was approved by the Ethics Committee of the School of Public Health, Shanghai Jiao Tong University. The health information was primarily hospital-specific daily counts of admissions, i.e., overall summarized data and stratified by age and sex subgroups without any individual identifiers. The need for informed consent was therefore waived by the institutional review board.

## Results

### Hospital Admissions for Stroke

We identified a total of 18,651 hospital admissions for stroke in four general hospitals in Shanghai between 2014 and 2016, namely, 15,554 admissions for ischemic stroke, 2,888 admissions for intracerebral hemorrhage stroke, and 209 admissions for subarachnoid hemorrhage. Table 1 summarizes the characteristics of daily hospital admissions for stroke. On average, in these four hospitals, there were 17 admissions for stroke per day over the study period. More than half of the patients (62.5%) were men and 53.8% were aged ≥65 years. The number of stroke admissions during cool days (9,250 admissions) was similar to the number of stroke admissions during warm days (9,341 admissions).

**Table 1:** Summary statistics of daily hospital admissions for stroke in four hospitals in Shanghai, 2014–2016.

**Characteristics**	**Total (*N*, %)**	**Mean**	**Standard deviation**	**Percentiles**			
				**P25**	**P50**	**P75**	**Max**
Stroke	18,651	17.0	5.52	13	17	21	40
Subtype of stroke							
Ischemic stroke	15,554 (83.4)	14.19	5.03	10	14	18	38
Intracerebral hemorrhage stroke	2,888 (15.5)	2.64	1.71	1	2	4	10
Subarachnoid hemorrhage	209 (1.1)	0.19	0.43	0	0	0	2
Sex							
Female	6,985 (37.5)	6.37	2.86	4	6	8	17
Male	11,666 (62.5)	10.64	3.92	8	10	13	26
Age							
<65 years	8,605 (46.1)	7.85	3.33	5	8	10	24
≥65 years	10,041 (53.9)	9.16	3.60	7	9	12	23
Apparent temperature							
Warm days	9,250 (49.6)	16.91	5.63	13	17	21	40
Cool days	9,341 (50.4)	17.11	5.43	13	17	21	34
Season							
Warm season	9,337 (50.1)	17.01	5.44	13	17	21	40
Cool season	9,314 (49.9)	17.03	5.61	13	17	21	34

*P25, the 25th percentile; P50, the 50th percentile; P75, the 75th percentile; Max, maximum. Total denotes the total number of hospital admissions during the study period; % denotes the percentage of admissions in the subgroup within the overall stoke admissions; and daily mean denotes the mean of daily hospital admissions (counts per day)*.

### Exposure Variables

**Figure 2:**
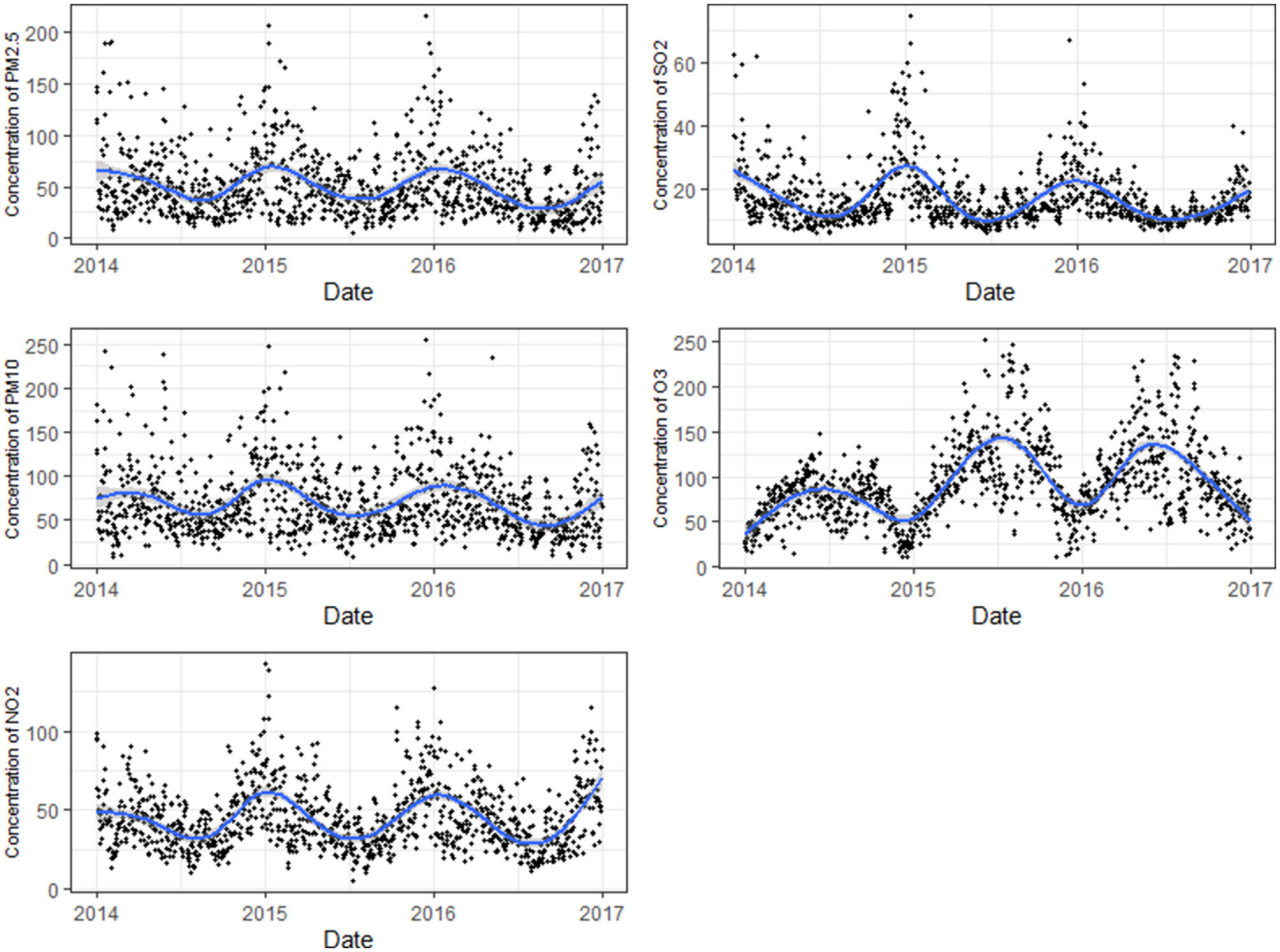
Concentrations of air pollutants in Shanghai during 2014–2016.

Table 2 presents the summary statistics of air pollutants and meteorological variables in Shanghai between 2014 and 2016. The average AT for Shanghai was 18.67°C and peaked at 40.02°C, which is slightly higher than a mean of 18.02°C and maximum of 34.5°C for ambient temperature. The wider range of ATs compared with the ambient temperature can be explained that including wind speed and humidity described the perceived thermal environment more correctly. The annual means (SD) of air pollutants were 50.18 (32.2) μg/m^3^ for PM_2.5_, 70.16 (39.24) μg/m^3^ for PM_10_, 44.29 (20.07) μg/m^3^ for NO_2_, 16.44 (9.01) μg/m^3^ for SO_2_, and 91.14 (43.2) μg/m^3^ for O_3_ (Figure 2). Supplementary Table 1 lists the summary statistics of 7-day average concentrations of air pollutants. On warm days (AT >19.56°C), the means of PM_2.5_, PM_10_, NO_2_, and SO_2_ concentrations were higher than in the means on cool days (AT ≤ 19.56°C), while the average levels of O_3_ on warm days were lower than on cool days.

**Table 2:** Summary statistics of air pollutants and meteorological variables in Shanghai, 2014–2016.

**Pollutants**	**Mean**	**Standard deviation**	**Min**	**Percentiles**			**Max**	**IQR**
				**P25**	**P50**	**P75**		
**All days**								
PM_2.5_	50.18	32.20	5	27	42	65	216	38
PM_10_	70.16	39.24	8	42	60	90.78	256	48.78
NO_2_	44.29	20.07	5	29.68	40	55	143	25.32
SO_2_	16.44	9.01	5.8	11	14	19	75	8
O_3_	91.94	43.20	10.7	62	85	115	253	53
**Cool days (AT** **<** **19.56°C)**								
PM_2.5_	59.55	36.97	8.20	32	50	79	216	45
PM_10_	81.65	43.70	8	49.75	70.60	108	216	58.25
NO_2_	53.08	21.35	13	37	50	66	143	29
SO_2_	20.37	10.69	6.9	13	17	24.03	75	11.03
O_3_	73.98	32.07	10.7	52	73	91.15	204	39.15
**Warm days (AT** **≥** **19.56°C)**								
PM_2.5_	40.92	23.19	5	23	36	53	145.2	30
PM_10_	58.78	30.25	8	37	51	73	238.6	36
NO_2_	35.40	13.85	2	25	33.6	43	99	18
SO_2_	12.52	4.21	5.8	10	11.5	14	36.2	4
O_3_	110.06	45.36	17.2	78	101	133	253	55
**Meteorological variables**								
Temp	18.02	8.39	−5.50	10.63	19.25	24.88	34.50	14.25
RH	73.75	12.46	35	66	75	83	98	17
AT	18.67	11.01	−9.01	9.03	19.56	27.45	40.02	18.42

*PM_2.5_, particulate matter with a diameter ≤ 2.5 μm; PM_10_, particulate matter with a diameter ≤ 10 μm; NO_2_, nitrogen dioxide; SO_2_, sulfur dioxide; O_3_, ozone; Temp, temperature; RH, relative humidity; AT, apparent temperature; P25, the 25th percentile; P50, the 50th percentile; P75, the 75th percentile; Max, maximum; Min, minimum; IQR, interquartile range*.

Table 3 lists Spearman's correlation coefficients for exposure variables. PM_2.5_ was highly correlated with PM_10_ (*r* = 0.92, *P* < 0.001). PM_2.5_, PM_10_, NO_2_, and SO_2_ were moderately correlated with each other (median *r* = 0.70, *P* < 0.001), while O_3_ levels were weakly negatively correlated with NO_2_ and SO_2_ and not correlated with PM_2.5_ (*r* = 0.02, *P* > 0.05) and PM_10_ (*r* = 0.06, *P* < 0.001). Ambient temperature, relative humidity, and AT were all negatively correlated with all air pollutants except O_3_.

**Table 3:** Spearman's correlation coefficients between exposure variables.

	**PM_**2.5**_**	**PM_**10**_**	**NO_**2**_**	**SO_**2**_**	**O_**3**_**	**Temp**	**RH**	**AT**
PM_2.5_	1	0.92^*^	0.68^*^	0.79^*^	0.02	−0.25^*^	−0.25^*^	−0.27^*^
PM_10_	0.92^*^	1	0.66^*^	0.75^*^	0.06^*^	−0.24^*^	−0.42^*^	−0.27^*^
NO_2_	0.68^*^	0.66^*^	1	0.70^*^	−0.26^*^	−0.43^*^	−0.19^*^	−0.44^*^
SO_2_	0.79^*^	0.75^*^	0.70^*^	1	−0.20^*^	−0.51^*^	−0.59^*^	−0.55^*^
O_3_	0.02	0.06^*^	−0.26^*^	−0.20^*^	1	0.45^*^	−0.13^*^	0.43^*^
Temp	−0.25^*^	−0.24^*^	−0.43^*^	−0.51^*^	0.45^*^	1	0.32^*^	0.99^*^
RH	−0.25^*^	−0.42^*^	−0.19^*^	−0.59^*^	−0.13^*^	0.32^*^	1	0.39^*^
AT	−0.27^*^	−0.27^*^	−0.44^*^	−0.55^*^	0.43^*^	0.99^*^	0.39^*^	1

*PM_2.5_, particulate matter with a diameter ≤ 2.5 μm; PM_10_, particulate matter with a diameter ≤ 10 μm; NO_2_, nitrogen dioxide; SO_2_, sulfur dioxide; O_3_, ozone; Temp, temperature; RH, relative humidity; AT, apparent temperature*.

* *P < 0.05*.

### Associations Between Air Pollutant Concentrations and Stroke Admissions

**Figure 3:**
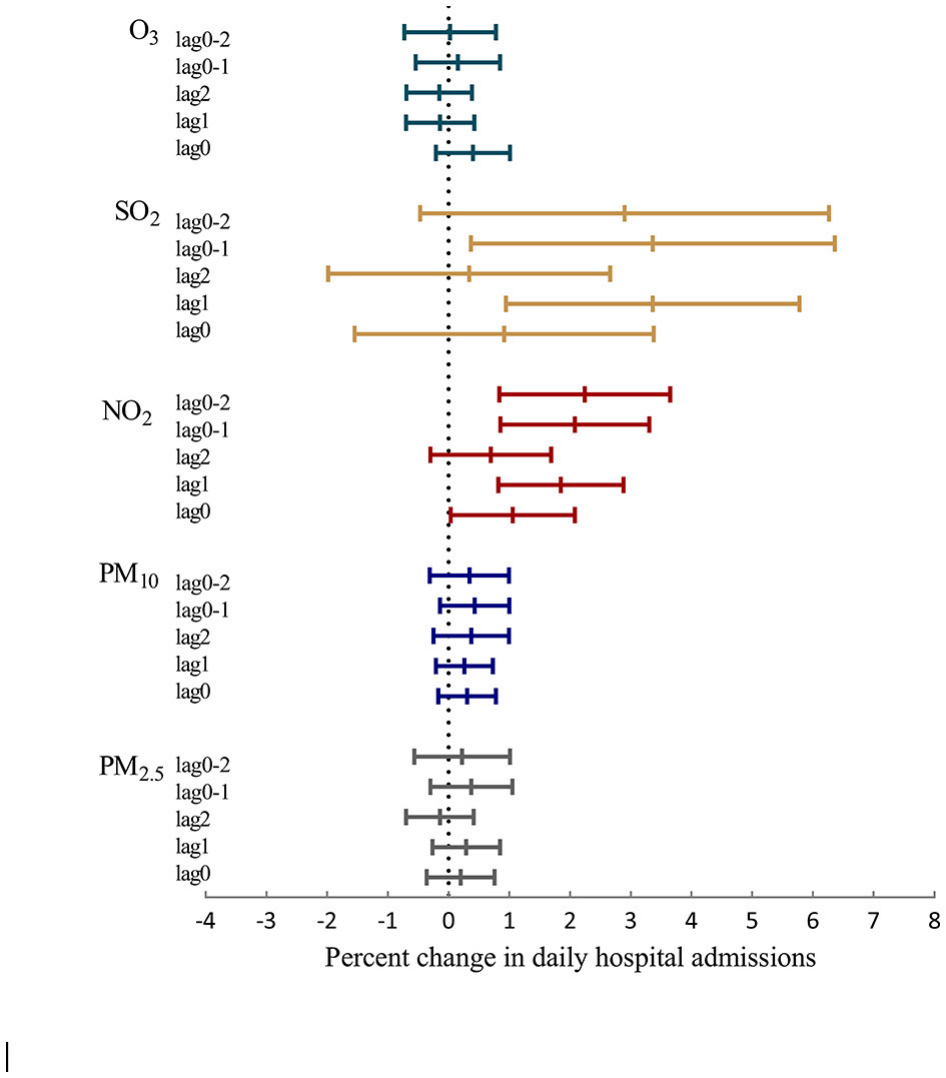
Percentage changes and 95% CIs in daily hospital admissions for stroke per 10 μg/m^3^ increase in PM_2.5_, PM_10_, NO_2_, SO_2_, and O_3_ concentrations at different lag days.

Figure 3 and Supplementary Table 2 present the percentage changes in hospital admissions for stroke with single-day lags of 0, 1, and 2 days and lags of 0–1 and 0–2 days associated with a 10 μg/m^3^ increase in the levels of the five air pollutants. Overall, NO_2_ and SO_2_ were consistently and significantly associated with hospital admissions for all types of stroke. Increases of 10 μg/m^3^ in concurrent-day PM_2.5_, PM_10_, SO_2_, NO_2_, and O_3_ levels corresponded to 0.2 (95% CI: −0.36%, 0.76%), 0.3% (95% CI: −0.17%, 0.78%), 1.05 (95% CI: 0.03%, 2.08%), 0.9 (95% CI: −1.53%, 3.39%), and 0.4% (95% CI: −0.2%, 1.01%) increases in hospital admissions for stroke, respectively. A significant lag effect of NO_2_ and SO_2_ exposure on stroke admissions was also observed. For NO_2_, the estimated effects were significant for same-day, prior-day, and moving average of lags 0–1 and 0–2 exposure, with the largest effect size observed for moving average of lag 0–2. Percent changes in the daily number of stroke admissions per 10 μg/m^3^ increase in NO_2_ ranged from 1.05 (95% CI: 0.82%, 2.88%) to 2.24% (95% CI: 0.84%, 3.65%). For SO_2_, the largest effect estimates were observed for a prior-day exposure. For each 10 μg/m^3^ increase in SO_2_ concentrations at lags 1, 2, 0–1, and 0–2, the RR of daily stroke admissions increased by 3.34 (95% CI: 0.955%, 5.79%), 0.32 (95% CI: −1.97%, 2.67%), 3.33 (95% CI: 0.38%, 6.37%), and 2.86% (95% CI: −0.45%, 6.28%), respectively. We did not observe significant lag effects for exposure to PM_2.5_, PM_10_, or O_3_. In the two-pollutant models, effect estimates for same-day exposure to NO_2_ were largely unchanged, but the effects of same-day exposure to SO_2_ became statistically insignificant when controlling for the effect of O_3_ (Table 4).

**Table 4:** Percent changes in daily hospital admissions for ischemic stroke associated with a 10 μg/m^3^ increase in air pollutant concentrations in two-pollutant models.

**Pollutants**	**Percent change**	**95% CI**
PM_2.5_ adjusted for O_3_	0.134	−0.435, 0.707
PM_10_ adjusted for O_3_	0.267	−0.212, 0.748
NO_2_ adjusted for O_3_	1.040^*^	0.022, 2.069
SO_2_ adjusted for O_3_	0.947	−1.488, 3.442
O_3_ adjusted for PM_2.5_	0.371	−0.247, 0.993
O_3_ adjusted for PM_10_	0.351	−0.262, 0.968
O_3_ adjusted for NO_2_	0.386	−0.220, 0.996
O_3_ adjusted for SO_2_	0.405	−0.203, 1.017

*PM_2.5_, particulate matter with a diameter ≤ 2.5 μm; PM_10_, particulate matter with a diameter ≤ 10 μm; NO_2_, nitrogen dioxide; SO_2_, sulfur dioxide; O_3_, ozone*.

* *P < 0.05*.

**Figure 4:**
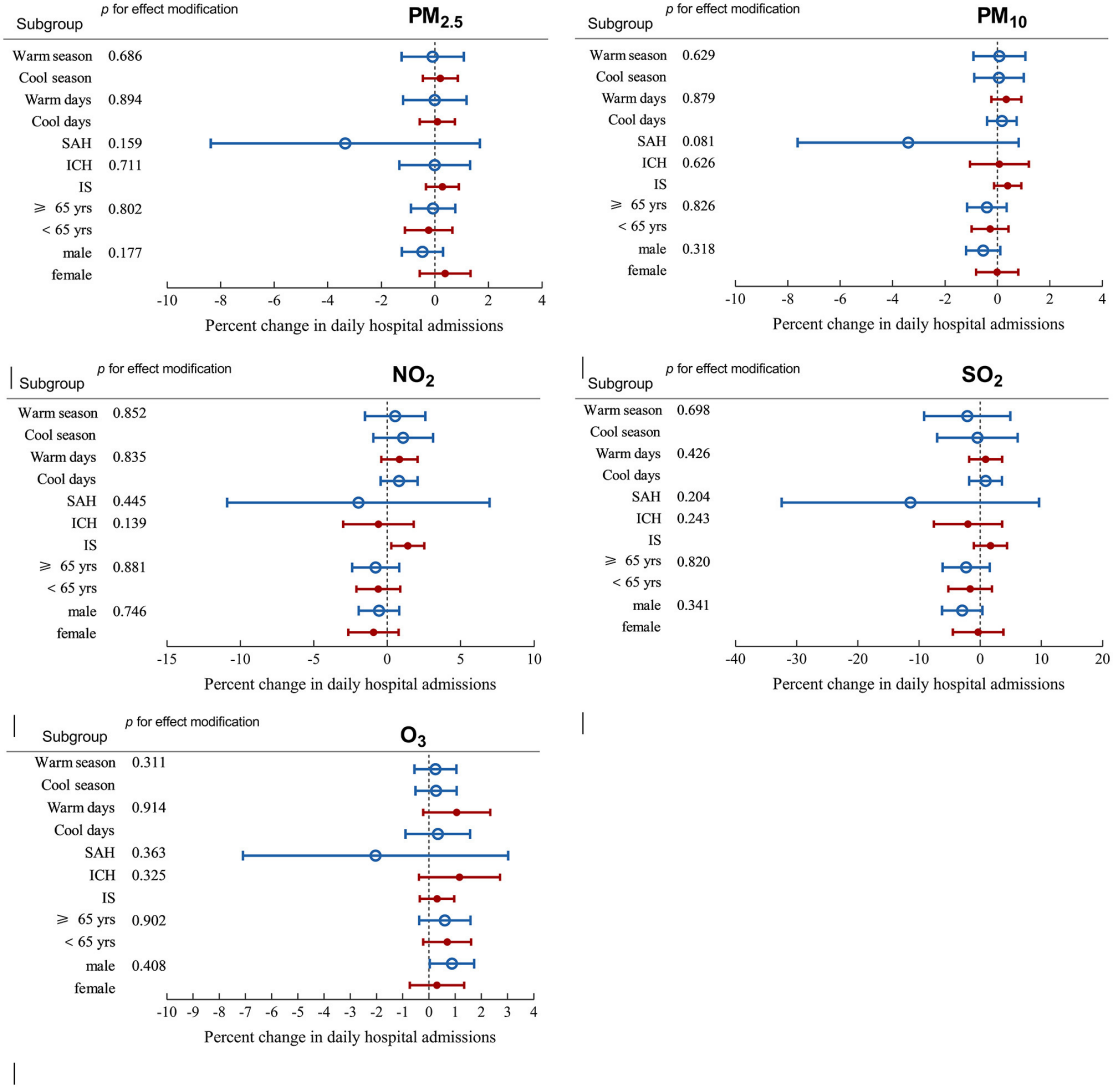
Percentage changes with 95% CIs in hospital admissions for stroke with 10 μg/m^3^ increase in particulate matter with a diameter ≤ 2.5 μm (PM_2.5_), particulate matter with a diameter ≤ 10 μm (PM_10_), nitrogen dioxide (NO_2_), sulfur dioxide (SO_2_), and ozone (O_3_) on lag 0 stratified by sex, age, apparent temperature, and season. The first column from the left in each panel indicates the subgroup and the second column indicates the *P* value generated from the *Z* test.

Figure 4 and Supplementary Table 3 show the associations between air pollutant levels (lag 0) and admissions for ischemic stroke stratified by sex, age, stroke subtype, AT, and season. We did not observe evidence for effect modification by these characteristics. Table 4 lists the results from the two-pollutant models.

## Discussion

Overall, our findings indicated that transient increases in SO_2_ and NO_2_ were associated with increased hospitalization for stroke in the single-pollutant models. The association between the NO_2_ levels and hospitalization for stroke remained significant in the two-pollutant models. However, we did not find evidence that the associations were modified by sex, age, season, or AT. Our findings add to the body of knowledge on the acute effects of air pollution on stroke in low- and middle-income countries. Although the magnitude of association appears to be relatively small, the public health burden derived from the risk could be substantial. The study could strengthen the rationale for reducing concentrations of air pollutants in megacities in low- and middle-income countries.

We found that prior 1- or 2-day exposure to NO_2_ and SO_2_ was positively associated with increased risk of hospital admission for stroke in the single-pollutant model, in general accordance with prior studies (6, 17, 29–33). However, the biological mechanisms underlying these perceived associations are unknown, and most studies have focused on the effects of particulate matter. Several potential mechanistic pathways have been proposed, namely, systemic inflammation (34, 35), thrombosis, (36–38) artery calcification (39), and vascular endothelial dysfunction (40). Some controlled studies have indicated that exposure to air pollutants (namely, NO_2_ and SO_2_) could adversely affect vascular endothelial function, increase systemic inflammation and platelet activation and the activity of the sympathetic nervous system, and decrease the activity of antioxidant enzymes. These effects may result in vasoconstriction, elevated blood pressure, ischemia, and risk of thrombosis (38, 41, 42). Another plausible mechanism of action is atrial arrhythmia, which may predispose patients to thromboembolic events (6).

Epidemiological studies of short-term exposure to air pollution and hospital admissions for stroke have generated inconclusive results (43). A 2021 systematic review and meta-analysis reported that 10 μg/m^3^ increases in the concentrations of all five pollutants were associated with hospital admissions for stroke; odds ratios ranged from 1.002 for O_3_ to 1.023 for NO_2_. However, these odds ratios are all very close to 1, indicating no association. Additionally, a meta-analysis published in 2015 observed robust and clear associations between both gaseous (RR 1.019 per 10 ppb increase in SO_2_, 95% CI: 1.011–1.027; RR 1.014 per 10 ppb increase in NO_2_, 95% CI: 1.009–1.019) and particulate air pollution (1.011 per 10 μg/m^3^ (1.011–1.012) for PM_2.5_ and 1.003 per 10 μg/m^3^ (1.002–1.004) for PM_10_). This study also noticed significant heterogeneity across pollutants that could be attributed to different study designs, different exposure lags, systemic differences in the baseline characteristics of the underlying population, misclassification of exposure measurement, and a different definition of stroke cases (6).

For example, a study conducted among individuals aged > 65 in the United States reported that interquartile range increases in same-day concentrations of PM_10_ (22.96 μg/m^3^), NO_2_ (11.93 μg/m^3^), and SO_2_ (6.96 ppb) concentrations that were associated with 1.03, 2.94, and 1.35% increases in admissions for ischemic stroke admissions, respectively (44). Epidemiological studies in numerous cities in China have also provided evidence of elevated risk of stroke or mortality associated with increases in daily concentrations of these air pollutants (8, 21, 29, 32, 45, 46). However, a study conducted in eight cities in France did not find an association between short-term exposure to air pollution and stroke (47). A nonsignificant increase in emergency hospital admissions for stroke related to particulate air pollutants was reported for one hospital in Taipei (46). Similarly, a case-crossover study in seven cities in Australia and New Zealand failed to observe associations between air pollutant levels and stroke among elderly individuals (48). The heterogeneity of results may be attributable to geographic variations, differences in pollutant concentrations, outcomes measured, population susceptibility, and the sources and constituents of the pollutants. For example, the greater adverse effects of PM_2.5_ and PM_10_ observed in other studies may be partly attributable to the relatively higher levels of particulate pollution at those study sites. According to Huang et al. (8) the median concentration of PM_2.5_ and PM_10_ in 2013–2014 in Beijing (a typical northern city in China) was 71.4 and 105.4 μg/m^3^, respectively, notably higher than the 50.2 and 70.2 μg/m^3^ in Shanghai (a typical southern city) in this study. Additionally, we only observed short-term associations of NO_2_ and SO_2_ with the risk of hospital admission for stroke; as the most densely populated city in China, pollution sources of Shanghai are mainly the gaseous emissions from motor vehicles, explaining why the effects of NO_2_ and SO_2_ were much more evident in our study than those of particulate pollutants.

We did not observe differences in the effect between warm days (AT > 19.6°C) and cool days (AT ≤ 19.6°C). Several studies have reported mixed results on the effect modification by temperature in air pollution-stroke associations. A study examining the associations between air pollutant levels and hospital admissions for transient ischemic attack reported that the effects of PM_2.5_, PM_10_, SO_2_, and O_3_ were more pronounced on warm days. Huang and colleagues reported that the positive associations of PM_2.5_ and PM_10_ with hospital admissions for both ischemic and hemorrhagic stroke were higher on warm days (>13.5°C) 0.8 Similar findings were also reported in studies of stroke admissions and cause-specific mortality in Taipei, Tianjin (China), and Canada (49–51). However, a study in Wuhan reported an association of NO_2_ with stroke only during the cold season (52). A national-scale in China reported a stronger association for ischemic stroke in the cool season than in the warm season (8). These findings perhaps indicated the combined effects of temperature, high levels of air pollution, and variations in ventilation conditions across seasons, or reflected seasonal differences in chemical compositions and toxicological characteristics of air pollutants (5, 8). In addition, stronger association on warm days could be explained that high ambient temperature may accelerate the emission, play an important role in determining transportation, dilution, chemical transformation of pollutants, and influence the eventual deposition. Besides, people tend to spend more time outdoors on warm days, resulting in more exposure to ambient air pollution (8, 17). Unlike the previous studies, which considered ambient temperature, we used AT to define warm/cool days. Combining temperature, humidity, and wind speed, AT can represent thermal comfort. Humidity conditions and wind could affect mechanisms of heat exchange to maintain homeostasis under heat-stress situations (53). The previous study reported that the air temperature alone seemed to overestimate the potential risks while the combination of air temperature, humidity, and wind speed adjusts the exposure to the thermal environment better (53, 54). Perspiration regulates body temperature, and high humidity may impair heat-exchange efficiency by reducing the rate of moisture evaporation from skin surfaces. At the same time, the convective rate is enhanced under windy conditions. However, we did not detect an effect modification by AT in associations between air pollution and stroke admissions, perhaps because of the relatively small number of admissions, the limited study duration, and the single-city study design. As a recent review suggested, the evaluation of the interaction between temperature and air quality and assessment of both on human health still retain some uncertainties (11). Thus, well-designed and multicenter studies with large samples are warranted to investigate these relationships.

There are several limitations to our study. First, the admission date may result in temporal misalignment between exposure and outcome, as the onset of stroke symptoms often began in the days before admission. Second, we used average city-wide concentrations of air pollutants rather than individual exposure, which may lead to exposure measurement errors and a more conservative conclusion. Third, the current study did not consider the direct association of AT with stroke outcomes because previous studies have suggested that temperature affects air pollution rather than the reverse (55). However, there may be synergistic effects of ambient temperature and air pollution on health (12). Therefore, further analysis is warranted to check the interaction between ambient air pollution and AT on health outcomes. Fourth, we dichotomized days as cool/warm days using median AT, which may have diluted the potential effect modification of temperature. Future studies with larger samples could classify days according to the distribution (percentiles) of temperature, which may assist in elucidating the effect modification. Finally, we used mean values of all monitoring sites as air pollution exposure levels and did not consider potential variations within the city, which might dilute the exposure levels in some districts with high levels of air pollution.

## Conclusions

We found that elevated levels of NO_2_ and SO_2_ were associated with an increase in hospital admissions for stroke, with a significant lag effect. Further research is warranted to determine whether AT modifies the associations between air pollution and admissions for stroke. This study adds to the available evidence in megacities in low- and middle-income countries and may promote the development of related public health policy.

## Data Availability Statement

The data analyzed in this study is subject to the following licenses/restrictions: The data on hospital admission was derived from hospitals, which cannot be public. Requests to access these datasets should be directed to kevinwlk21@126.com.

## Ethics Statement

The studies involving human participants were reviewed and approved by Ethics Committee of School of Public Heath Shanghai Jiao Tong University. The ethics committee waived the requirement of written informed consent for participation.

## Author Contributions

Z-JZ and LW: conceptualization and validation. NL: methodology, software, formal analysis, and visualization. TF: investigation. RZ: resources. TF and RZ: data curation. LW: original draft preparation. Z-JZ and NL: review and editing. Z-JZ: supervision. All authors have read and agreed to the published version of the manuscript.

## Conflict of Interest

The authors declare that the research was conducted in the absence of any commercial or financial relationships that could be construed as a potential conflict of interest.

## Publisher's Note

All claims expressed in this article are solely those of the authors and do not necessarily represent those of their affiliated organizations, or those of the publisher, the editors and the reviewers. Any product that may be evaluated in this article, or claim that may be made by its manufacturer, is not guaranteed or endorsed by the publisher.

## Abbreviations

CI: confidence interval
*df*: degree of freedom
NO_2_: nitrogen dioxide
PM_2._5: particulate matter with a diameter ≤ 2.5 μm
PM_10_: particulate matter with a diameter ≤ 10 μm
SO_2_: sulfur dioxide.
