# Supplementary material for: Short-Term Association of Air Pollutant Levels and Hospital Admissions for Stroke and Effect Modification by Apparent Temperature: Evidence From Shanghai, China
Source: Front Public Health. 2021 Sep 27;9:716153. doi: 10.3389/fpubh.2021.716153 (PMC8503471; doi:10.3389/fpubh.2021.716153)
Supplement: Supplementary file 1 [file Data_Sheet_1.docx]

Table S1 Descriptive statistics of 7-day average concentrations of air pollutants

| Pollutants | Mean | Standard Deviation | Minimum | Maximum | Range |
| --- | --- | --- | --- | --- | --- |
| PM_2.5_ | 50.17 | 20.34 | 15.43 | 121.29 | 105.86 |
| PM_10_ | 70.15 | 24.80 | 28.71 | 141.71 | 113.00 |
| SO_2_ | 16.45 | 6.82 | 8.14 | 40.86 | 32.71 |
| NO_2_ | 44.35 | 14.55 | 15.00 | 78.57 | 63.57 |
| O_3_ | 91.82 | 36.26 | 21.49 | 219.86 | 198.37 |

Table S2 Percentage changes and 95% confidence intervals in daily hospital admissions for stroke per 10 μg/m^3^ increase in PM_2.5_, PM_10_, NO_2_, SO_2_, O_3_ concentrations at different lag days

| Exposure day | Percent change | 95% lower limit | 95% upper limit |
| --- | --- | --- | --- |
| **PM_2.5_** |  |  |  |
| lag 0 | 0.198 | -0.361 | 0.761 |
| lag 1 | 0.289 | -0.267 | 0.847 |
| lag 2 | -0.144 | -0.698 | 0.414 |
| lag 0-1 | 0.373 | -0.300 | 1.050 |
| lag 0-2 | 0.221 | -0.564 | 1.013 |
| **PM_10_** |  |  |  |
| lag 0 | 0.304 | -0.170 | 0.781 |
| lag 1 | 0.261 | -0.206 | 0.731 |
| lag 2 | 0.371 | -0.247 | 0.993 |
| lag 0-1 | 0.428 | -0.142 | 1.000 |
| lag 0-2 | 0.341 | -0.310 | 0.997 |
| **NO_2_** |  |  |  |
| lag 0 | 1.053 | 0.034 | 2.082 |
| lag 1 | 1.846 | 0.822 | 2.880 |
| lag 2 | 0.692 | -0.296 | 1.691 |
| lag 0-1 | 2.074 | 0.858 | 3.305 |
| lag 0-2 | 2.236 | 0.839 | 3.653 |
| **SO_2_** |  |  |  |
| lag 0 | 0.897 | -1.534 | 3.389 |
| lag 1 | 3.344 | 0.955 | 5.790 |
| lag 2 | 0.321 | -1.974 | 2.669 |
| lag 0-1 | 3.335 | 0.384 | 6.372 |
| lag 0-2 | 2.863 | -0.448 | 6.285 |
| **O_3_** |  |  |  |
| lag 0 | 0.398 | -0.209 | 1.010 |
| lag 1 | -0.140 | -0.700 | 0.424 |
| lag 2 | -0.154 | -0.691 | 0.386 |
| lag 0-1 | 0.150 | -0.543 | 0.849 |
| lag 0-2 | 0.022 | -0.729 | 0.780 |

Table S3 Percentage changes with 95% confidence intervals in hospital admissions for stroke with 10 μg/m^3^ increase in PM_2.5_, PM_10_, NO_2_, SO_2_ and O_3_ on lag 0 days stratified by sex, age, apparent temperature, and season

| Subgroups | Percent change | 95% lower limit | 95% upper limit | *p*-value from *Z* test |
| --- | --- | --- | --- | --- |
| **PM_2.5_** |  |  |  |  |
| female | 0.38 | -0.57 | 1.33 | 0.18 |
| male | -0.47 | -1.23 | 0.30 |  |
| ≥ 65 years | -0.07 | -0.90 | 0.76 | 0.80 |
| < 65 years | -0.24 | -1.12 | 0.66 |  |
| IS | 0.28 | -0.34 | 0.89 |  |
| ICH | -0.01 | -1.32 | 1.32 | 0.71 |
| SAH | -3.43 | -8.33 | 1.72 | 0.16 |
| Cool days | 0.09 | -0.56 | 0.75 | 0.89 |
| Warm days | -0.01 | -1.19 | 1.18 |  |
| Cool season | 0.20 | -0.45 | 0.86 | 0.69 |
| Warm season | -0.09 | -1.24 | 1.08 |  |
| PM_10_ |  |  |  |  |
| female | -0.01 | -0.82 | 0.79 | 0.32 |
| male | -0.55 | -1.20 | 0.11 |  |
| ≥ 65 years | -0.28 | -0.98 | 0.42 | 0.83 |
| < 65 years | -0.41 | -1.16 | 0.35 |  |
| IS | 0.39 | -0.13 | 0.91 |  |
| ICH | 0.07 | -1.04 | 1.20 | 0.63 |
| SAH | -3.47 | -7.59 | 0.84 | 0.08 |
| Cool days | 0.17 | -0.39 | 0.73 | 0.88 |
| Warm days | 0.07 | -0.92 | 1.07 |  |
| Cool season | 0.34 | -0.23 | 0.91 | 0.63 |
| Warm season | 0.05 | -0.89 | 1.00 |  |
| **NO_2_** |  |  |  |  |
| female | -0.95 | -2.65 | 0.78 | 0.75 |
| male | -0.56 | -1.94 | 0.83 |  |
| ≥ 65 years | -0.62 | -2.10 | 0.89 | 0.88 |
| < 65 years | -0.80 | -2.39 | 0.82 |  |
| IS | 1.39 | 0.28 | 2.52 |  |
| ICH | -0.62 | -2.99 | 1.81 | 0.14 |
| SAH | -2.23 | -10.75 | 7.10 | 0.45 |
| Cool days | 0.80 | -0.44 | 2.06 | 0.84 |
| Warm days | 0.53 | -1.50 | 2.60 |  |
| Cool season | 0.83 | -0.41 | 2.07 | 0.85 |
| Warm season | 1.07 | -0.94 | 3.12 |  |
| **SO_2_** |  |  |  |  |
| female | -0.39 | -4.44 | 3.83 | 0.34 |
| male | -2.98 | -6.21 | 0.36 |  |
| ≥ 65 years | -1.69 | -5.19 | 1.94 | 0.82 |
| < 65 years | -2.34 | -6.10 | 1.57 |  |
| IS | 1.65 | -1.02 | 4.41 |  |
| ICH | -2.10 | -7.52 | 3.64 | 0.24 |
| SAH | -13.08 | -31.56 | 10.40 | 0.20 |
| Cool days | 0.86 | -1.78 | 3.57 | 0.43 |
| Warm days | -2.28 | -9.08 | 5.02 |  |
| Cool season | 0.88 | -1.78 | 3.61 | 0.70 |
| Warm season | -0.60 | -6.95 | 6.18 |  |
| **O_3_** |  |  |  |  |
| female | 0.30 | -0.73 | 1.34 | 0.41 |
| male | 0.88 | 0.03 | 1.73 |  |
| ≥ 65 years | 0.69 | -0.21 | 1.61 | 0.90 |
| < 65 years | 0.60 | -0.37 | 1.58 |  |
| IS | 0.31 | -0.35 | 0.97 |  |
| ICH | 1.16 | -0.38 | 2.72 | 0.32 |
| SAH | -2.12 | -7.05 | 3.07 | 0.36 |
| Cool days | 0.34 | -0.89 | 1.58 | 0.91 |
| Warm days | 0.25 | -0.55 | 1.05 |  |
| Cool season | 1.05 | -0.22 | 2.34 | 0.31 |
| Warm season | 0.27 | -0.51 | 1.06 |  |
